# Head/skull injury potential of empty 0.5-l beer glass bottles vs. 0.33-l Coke bottles

**DOI:** 10.1007/s00414-021-02562-7

**Published:** 2021-03-30

**Authors:** C. Nentwig, S. Steinhoff, J. Adamec, S. N. Kunz

**Affiliations:** 1 Ulm University, Ulm, Germany; 2 Radiology Department of the German Armed Forces Hospital Ulm, Ulm, Germany; 3 Institute of Forensic Medicine, Ludwig-Maximilian University Munich, Munich, Germany; 4 Institute of Forensic Medicine, Ulm University Hospital, Ulm, Germany

**Keywords:** Forensic medicine, Glass bottles, Head injury, Assault, Blast with an object

## Abstract

The medical and biomechanical assessment of injuries from blows to the head is a common task in forensic medicine. In the context of a criminal justice process, the injury potential of different striking weapons is important. The article at hand compares the injury potential of assaults with a 0.5-l beer bottle and a 0.33-l Coke bottle, both made of glass. The research team hit 30 used empty 0.5-l beer bottles and 20 used empty 0.33-l Coke bottles manually on an aluminum dummy skull set on a force measuring plate, using acrylic and pork rind as a scalp surrogate. There was no significant difference in fracture threshold and energy transfer between the examined beer and Coke bottles. Both glass bottles are able to cause fractures to the facial bones while cranial bone fractures are primarily not to be expected. Blows with a 0.5-l beer bottle or with a 0.33-l Coke bottle to the head can transfer up to 1.255 N and thus are able to cause severe blunt as well as sharp trauma injuries.

## Introduction

The forensic-medical and biomechanical assessment of blunt and sharp force trauma injuries due to assaults are a common task for the practicing forensic specialist. Head trauma in particular is a complex area of study as it has many individual varieties in anatomy and mechanical properties. In this context, the injury potential of the striking weapon used is an essential part in the medicolegal analysis. The differentiation between minor and grievous bodily harm is based on the assessment of both the action performed and the weapon used. Fatal intracranial injuries are not necessarily accompanied by fractures to the skull. And while skull fractures per se are not life-threatening, they are often associated with complications (in particular with intracranial bleeding and secondary consequences such as inflammatory reactions) that can lead to death. For this reason, any impact to the head likely to cause a calvarium fracture can be considered potentially life-threatening.

To simplify and unite possible life-threatening blast scenarios to the head, in the current experimental analysis, the main fatal injury criterion of head trauma was limited to skull fractures. Thus, the experimentally established fracture thresholds of human skull bones were defined as the differentiation criterion between a potentially life-threatening and a non-lethal force.

This article evaluates the injury potential of 0.5-l and 0.33-l glass bottles when hit with its side aspect over the top of the human head and presents the key variables that can significantly influence a forensic-biomechanical evaluation of such action. We chose beer and Coke bottles because those beverages are very common and the glasses readily available as striking weapons.

## Methods

**Fig. 1:**
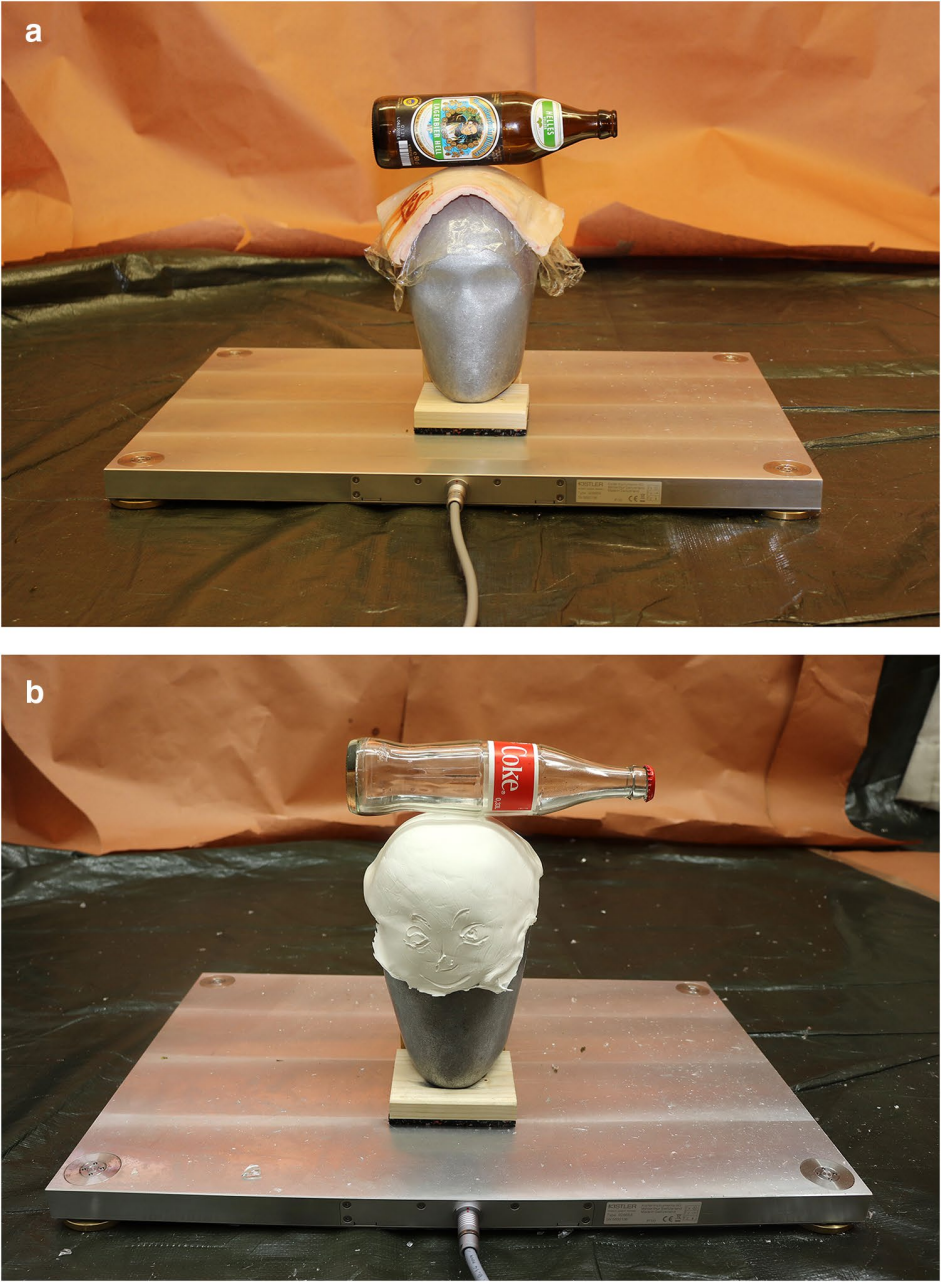
**a** Test setup used 0.5-l beer bottles. **b** Test setup used 0.33-l Coke bottles

We performed a series of trials with an aluminum dummy skull hybrid III covered with acrylic scalp surrogate (3 mm) or pork rind (5 mm) placed on a multi-component force measuring plate type 9286 B by Kistler Instrumente AG (Fig. 1). We recorded the floor reaction force in all three dimensions using the BioWare® software. The maximum force was set at 12 kN. Photos were taken with a Canon EOS 250D digital camera.

**Fig. 2:**
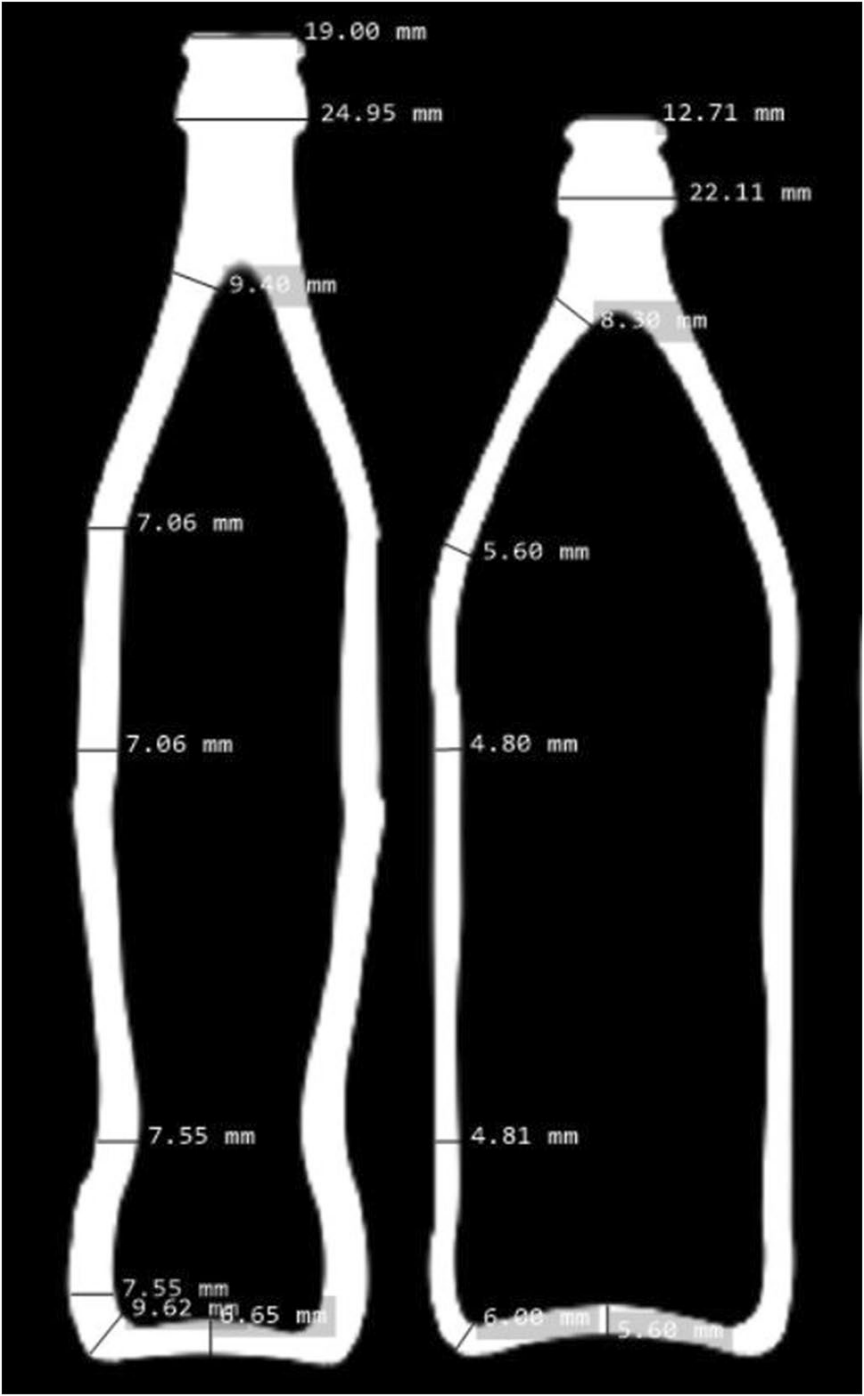
Axial slices of a CT scan of the used 0.5-l and 0.33-l glass bottles

Thirty used empty 0.5-l beer bottles from the Augustiner brewery (Euro Bottle, weight: 345 g) and 20 used empty 0.33-l Coke bottles from the Coca Cola Company (weight: 388 g) made of glass (Fig. 2) were manually hit onto the skull. Five female and two male volunteers performed the blows alternately in a vertical movement. The contact area was the cylindrical side surface of the bottles with the apex region of the dummy head. If the bottle remained intact, the experiment was carried out again with (subjectively) increased intensity and constant movement until the bottle broke. The aim was to detect the maximum possible force transmission to the skull. In addition, changes to the scalp surrogates and the fracture pattern of the bottles were analyzed.

## Results

Eighty-eight strikes were performed with 0.5-l beer bottles and 74 strikes with 0.33-l Coke bottles. Until the moment of fracture, none of the bottles showed visible signs of mechanical damage.

In general, the maximum impact force was not measured in the strikes that lead to bottle fracture.

The maximum force transfer of an unbroken 0.5-l beer bottle (1209 N) was slightly lower than the maximum of an unbroken 0.33-l Coke bottle (1255 N). The blows with empty glass bottles on the skull coated with acrylic and on the dummy head covered with pork rind showed comparable results. An average fracture force of 605 N (318–952 N) was found after 30 tests with beer bottles (Tables 1 and 2) and of 804 N (498–1220 N) after 20 tests with Coke bottles (Tables 3 and 4).

**Table 1:** Measurement results, strikes with the side of 0.5 l beer bottles on acrylic surrogate - aluminum

Acrylic scalp surrogate			
Nr	Fracture	Force (N)	Weight bottle/weight fragments
**1.1**	n	520.74	
1.2	n	606.82	
1.3	yes	**531.92**	
**2.1**	n	916.32	
2.2	n	877.52	
2.3	yes	**921.76**	
**3**	yes	**368.26**	
**4.1**	n	817.50	
4.2	n	987.42	
4.3	yes	**883.53**	
**5.1**	n	836.84	
5.2	yes	**669.87**	
**6**	yes	**342.86**	
**7.1**	n	671.75	
7.2	n	826.30	
7.3	n	766.39	
7.4	n	815.13	
7.5	n	933.96	
7.6	n	1003.59	
7.7	n	954.12	
7.8	yes	**838.92**	
**8**	yes	**318.85**	353 g
**9**	yes	**439.98**	351 g
**10**	yes	**494.90**	359 g
			69 g neck
			58 g label
			16 g bottom
			15 g fragment
**11.1**	n	607.90	
11.2	yes	**460.66**	73 g neck
			23 g fragment
			15 g fragment
**12**	yes	**386.84**	
**13**	yes	**369.67**	97 g neck
			25 g bottom
**14**	yes	**558.25**	73 g neck
			32 g bottom
			15 g fragment
**15**	yes	**464.49**	50 g neck
			50 g bottom
			17 g fragment
**16**	yes	**518.45**	36 g neck
			27 g fragment
**17**	yes	**405.51**	107 g label
			61 g neck
			19 g fragment
**18.1**	n	143.12	
18.2	n	311.65	
18.3	n	377.62	
18.4	n	472.07	
18.5	yes	**518.32**	214 g neck + label
**19.1**	n	778.65	
19.2	n	773.55	
19.3	n	429.23	
19.4	n	810.18	
19.5	n	779.63	
19.6	n	812.20	
19.7	n	860.72	
19.8	yes	**632.92**	73 g label
			69 g neck
			16 g fragment
**20.1**	n	598.41	
20.2	n	813.82	
20.3	n	358.15	
20.4	n	877.44	
20.5	yes	**521.23**	56 g label
			39 g neck

The bold entries represent the energy, which was transmitted when the bottles fractured. The letter n stands for “no” as in no fracture

**Table 2:** Measurement results, strikes with the side of 0.5 l beer bottles on pork rind - aluminum

Pork rind			
Nr	Fracture	Force (N)	Weight bottle/weight fragments
21.2	n	832.98	
21.3	n	850.69	
21.4	n	912.87	
21.5	n	1025.44	
21.6	n	900.11	
21.7	n	1057.09	
21.8	yes	**951.87**	56 g label
			28 g neck
			26 g fragment
			17 g fragment
**22**	yes	**394.02**	180 g neck + label
			38 g bottom
			19 g fragment
**23.1**	n	586.57	
23.2	n	868.22	
23.3	n	1131.83	
23.4	yes	**885.70**	95 g label
			35 g neck
			39 g bottom
			30 g neck-fragment
**24.1**	n	835.39	
24.2	n	863.84	
24.3	n	994.67	
24.4	yes	**827.58**	93 g neck
			83 g label
			21 g bottom
**25**	yes	**845.24**	103 g label
			40 g neck
			27 g fragment
			22 g bottom
**26.1**	n	622.17	
26.2	n	837.01	
26.3	yes	**478.15**	105 g neck
			43 g label
			41 g bottom
**27.1**	n	544.72	
27.2	n	580.88	
27.3	n	528.37	
27.4	n	881.06	
27.5	n	1001.06	
27.6	n	1208.60	
27.7	n	943.91	
27.8	yes	**850.76**	117 g label
			32 g neck
			27 g bottom
**28**	yes	**795.80**	80 g label
			79 g neck
			40 g bottom
**29.1**	n	895.09	
29.2	n	498.81	
29.3	n	936.39	
29.4	n	599.26	
29.5	n	892.51	
29.6	n	1168.50	
29.7	n	763.47	
29.8	yes	**903.10**	90 g neck
			50 g label
			38 g bottom
**30**	yes	**576.12**	119 g neck + label
			15 g bottom
			13 g fragment

The bold entries represent the energy, which was transmitted when the bottles fractured. The letter n stands for “no” as in no fracture

**Table 3:** Measurement results, strikes with the side of 0.33 l Coke bottles on pork rind—aluminum

Pork rind			
Nr	Fracture	Force (N)	Weight bottle/weight fragments
**1.1**	n	929.87	
1.2	n	1110.84	
1.3	n	1187.13	
1.4	n	1056.67	
1.5	yes	**1196.03**	56 g neck
			56 g back label
			24 g bottom
			22 g front label
**2.1**	n	811.07	
2.2	n	1099.50	
2.3	n	1255.32	
2.4	n	1230.40	
2.5	n	1144.87	
2.6	yes	**1131.72**	57 g label
			38 g neck
			16 g bottom
**3.1**	n	1082.43	
3.2	n	1106.75	
3.3	n	942.04	
3.4	n	1211.97	
3.5	yes	**1134.94**	44 g neck
			36 g back label
			25 g front label
			17 g bottom
**4.1**	n	1082.52	
4.2	n	853.66	
4.3	n	863.82	
4.4	n	884.57	
4.5	yes	**1219.87**	114 g neck
			39 g front label
			34 g bottom
**5**	yes	**1217.59**	54 g back label
			34 g neck
			25 g bottom
			19 front label
**6.1**	n	784.71	
6.2	n	839.91	
6.3	n	831.06	
6.4	n	823.84	
6.5	n	1221.86	
6.6	n	991.73	
6.7	yes	**994.61**	60 g front label
			40 g back label
			29 g neck
			14 g bottom
**7.1**	n	888.69	
7.2	n	1082.73	
7.3	yes	**850.87**	175 g neck + label
			41 g bottom
**8.1**	n	975.61	
8.2	n	1219.86	
8.3	n	1210.27	
8.4	yes	**718.39**	78 g neck
			14 g bottom
			14 g back label
			13 g front label
**9.1**	n	1154.62	
9.2	n	842.10	
9.3	n	801.71	
9.4	n	814.99	
9.5	yes	**660.19**	211 g neck + label
			19 g bottom
**10.1**	n	1224.07	
10.2	yes	**497.50**	74 g neck
			59 g back label
			34 g bottom
			12 g front label
**11.1**	n	786.07	
11.2	yes	**761.97**	180 g neck + label
			23 g bottom

The bold entries represent the energy, which was transmitted when the bottles fractured. The letter n stands for “no” as in no fracture

**Table 4:** Measurement results, strikes with the side of 0.33-l Coke bottles on acryl skin—aluminum

Acrylic scalp surrogate			
Nr	Fracture	Force (N)	Weight bottle/weight fragments
**12.1**	n	635.29	
12.2	yes	**614.15**	69 g back label
			44 g neck
			36 g front label
			20 g bottom
**13.1**	n	675.03	
13.2	n	962.99	
13.3	yes	**741.37**	56 g back label
			35 g neck
			28 g bottom
			20 g front label
**14.1**	n	809.36	
14.2	n	689.72	
14.3	n	809.03	
14.4	n	847.74	
14.5	n	983.64	
14.6	n	984.13	
14.7	yes	**592.83**	116 g neck + back label
			54 g front label
			9 g bottom
**15.1**	n	676.48	
15.2	n	730.67	
15.3	yes	**681.10**	108 g neck + back label
			24 g front label
			14 g bottom
**16**	yes	**534.63**	49 g neck
			39 g back label
			14 g bottom
**17**	yes	**520.08**	56 g neck
			43 g front label
			21 g back label
			17 g bottom
**18.1**	n	608.89	
18.2	n	554.14	
18.3	n	588.43	
18.4	yes	**544.26**	164 g neck + label
			22 g bottom
**19**	yes	**674.18**	104 g neck + front label
			39 g bottom
			28 g back label
**20.1**	n	740.18	
20.2	n	876.48	
20.3	n	850.66	
20.4	n	878.02	
20.5	n	846.83	
20.6	n	772.30	
20.7	yes	**794.88**	47 g neck
			29 g bottom
			26 g back label
			21 g front label

The bold entries represent the energy, which was transmitted when the bottles fractured. The letter n stands for “no” as in no fracture

**Fig. 3:**
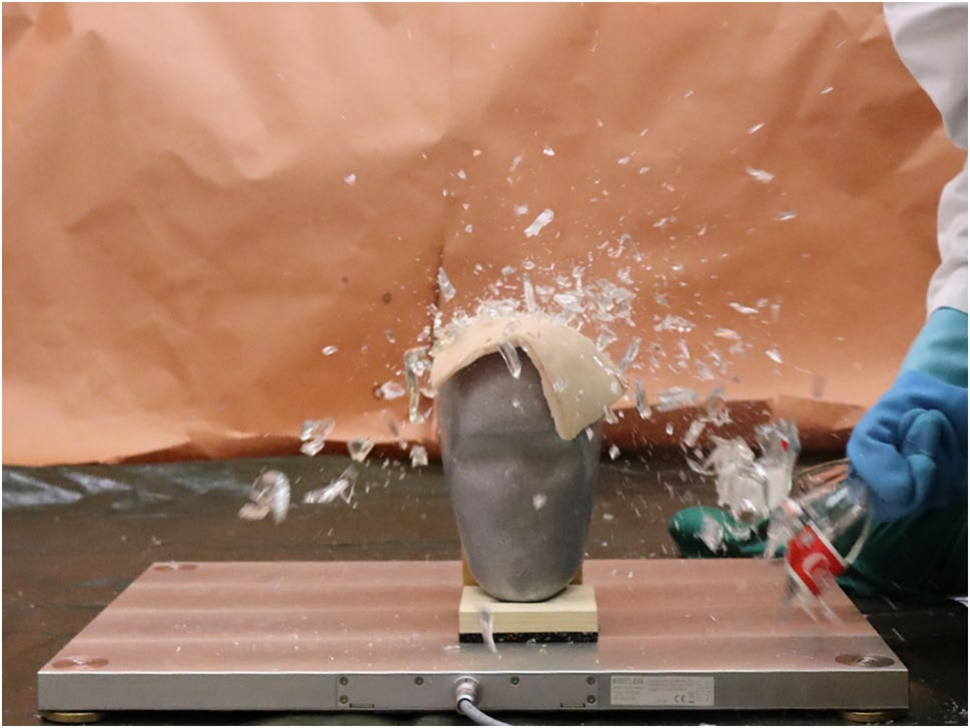
Fracturing 0.33-l Coke bottle when hitting the skull

**Fig. 4:**
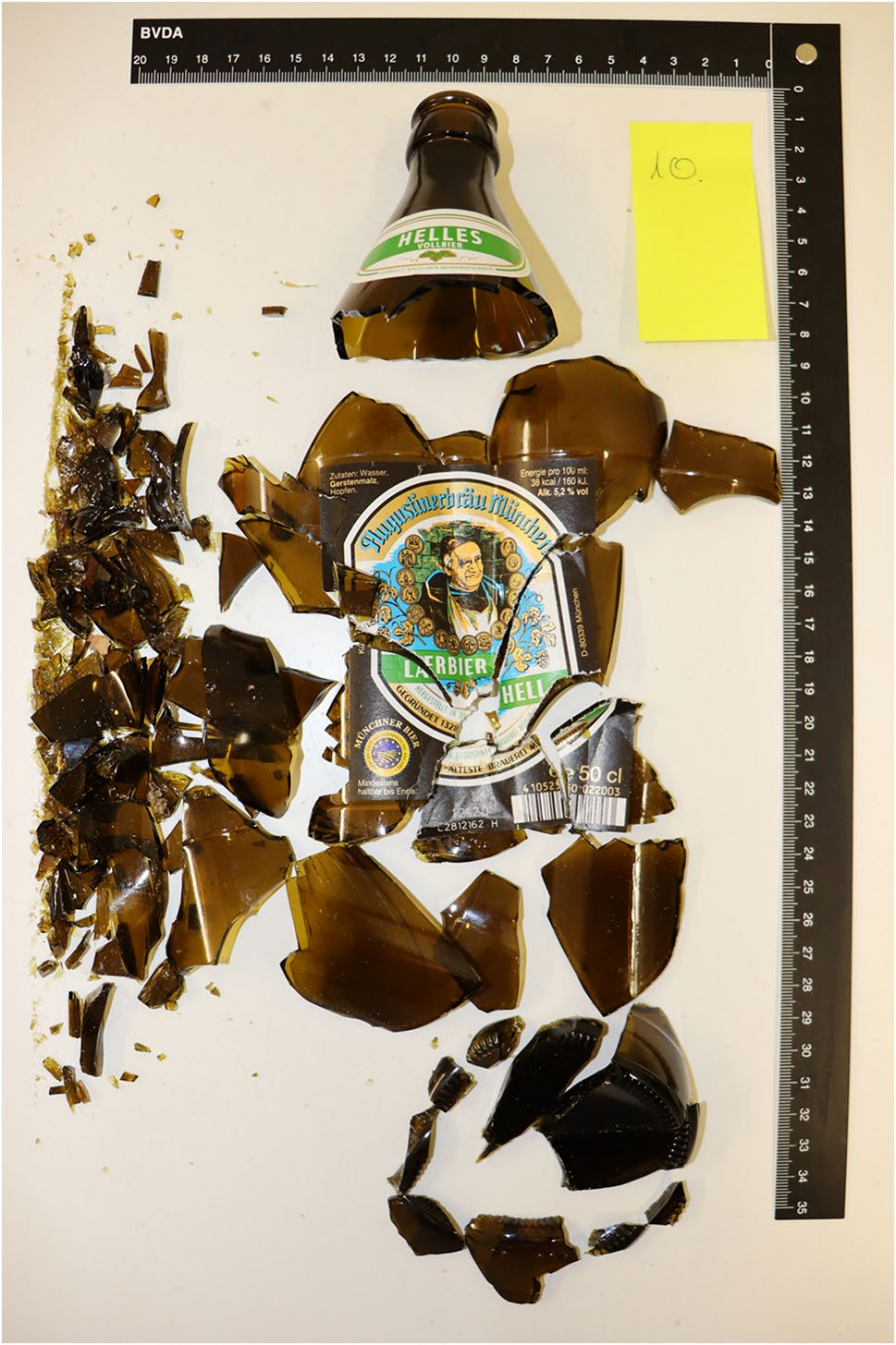
Glass fragments of a fractured 0.5-l beer bottle

**Fig. 5:**
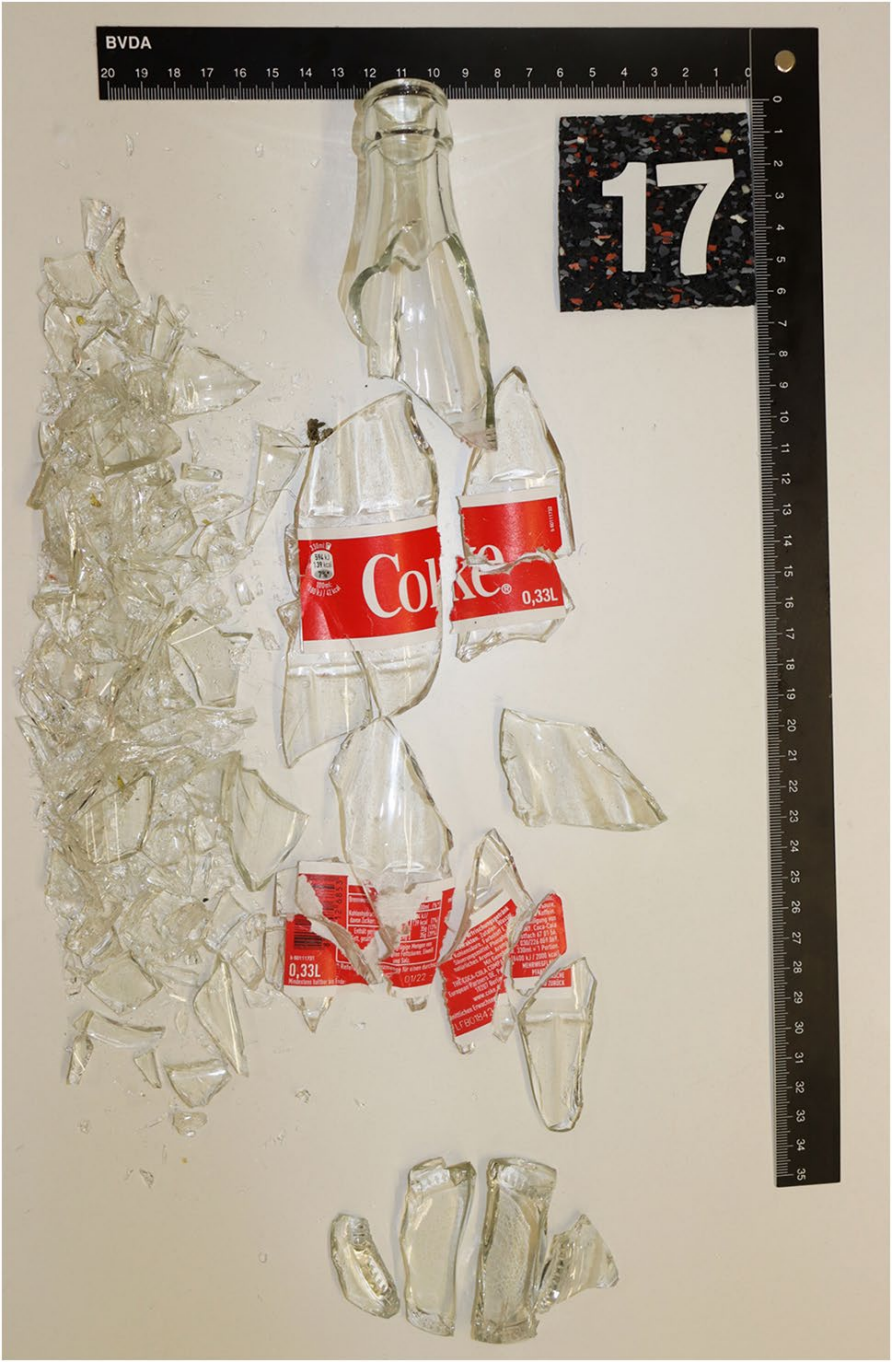
Glass fragments of a fractured 0.33-l Coke bottle

Both types of glass bottles fractured into many small individual pieces (> 30) while the upper approx. 3–6 cm of the bottle neck held with the hand remained intact (Figs. 3, 4 and 5). In the area of the label, the broken glass was usually held together as a composite. This area was slightly larger with the beer bottles. The largest fractured piece weighed 214 g (beer bottles) and 211 g (Coke bottles). A characteristic fracture behavior in the sense of traceable fracture lines or typical fracture pattern could not be determined (Figs. 4 and 5).

**Fig. 6:**
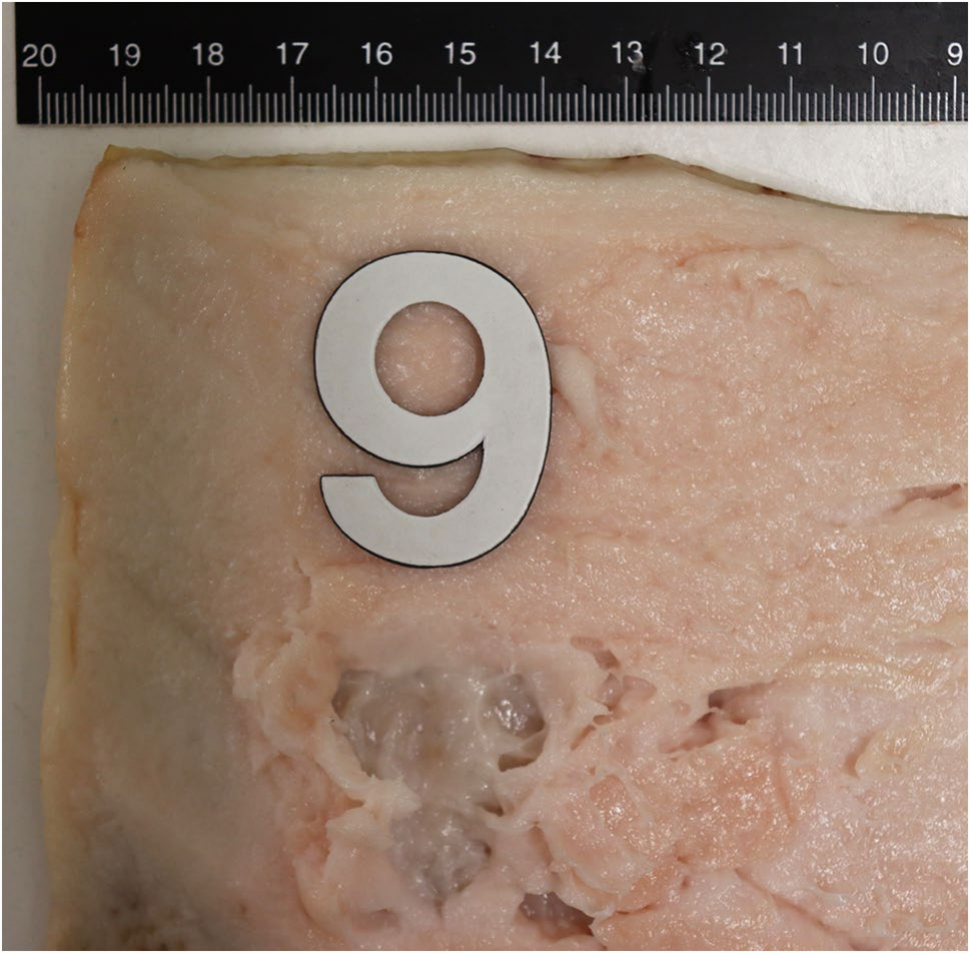
Blast injuries in the pork rind after strike with a 0.33-l Coke bottle

Both the acrylic skin and the pork rind showed soft tissue compression in the area of the contact point in almost every attempt (Fig. 6). No distinct differences could be detected in injury patterns resulting from strikes with a 0.5-l and a 0.33-l bottle. Sharp injuries were found on the pork rind in 5 attempts. These were superficial and small with a maximum length of 0.7 cm long and up to 2 mm deep.

**Fig. 7:**
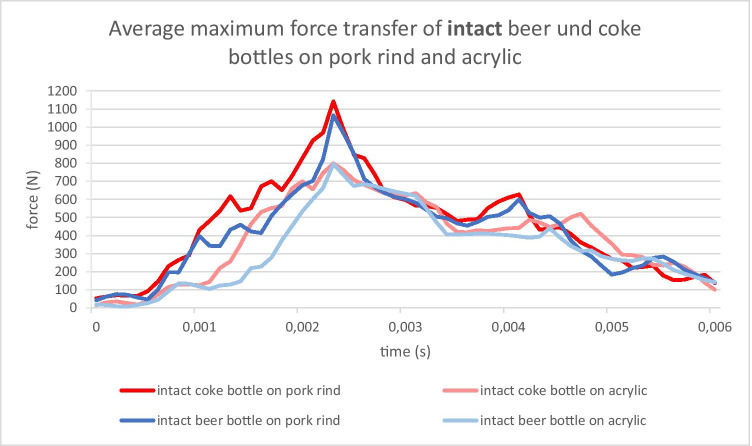
Force transfer over time during a strike with a 0.5-l beer bottle and a 0.33-l Coke bottle, intact

**Fig. 8:**
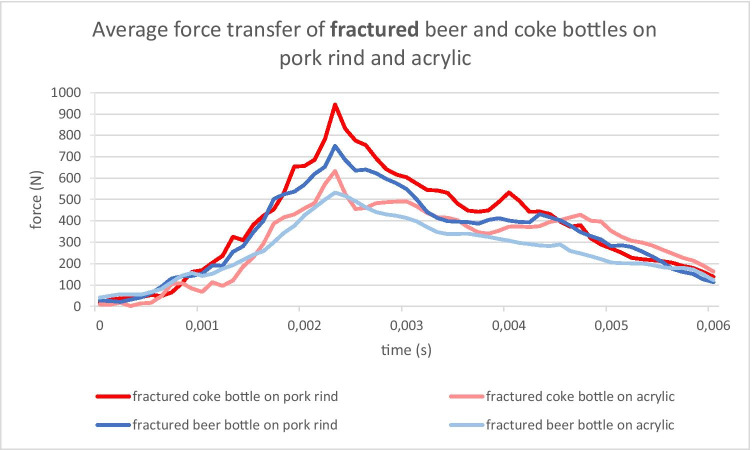
Force transfer over time during a strike with a 0.5-l beer bottle and a 0.33-l Coke bottle, fractured

Regardless of the type of bottle, the typical contact duration between the bottle and the target was between 4 and 5 ms. Thus, the energy transfer took place during an extremely short period of time. The force-over-time curves are shown as an example in Figs. 7 and 8 for intact and broken bottles, respectively.

## Discussion

The aim of our study was to gain new data regarding the mechanical properties of glass bottles that can then be used in criminal justice process. In case of a blow with a beer or a Coke bottle made of glass, the maximum force transmission is the parameter that matters most. When exceeding the biomechanical tolerance limit of the particular tissues, injuries of the skin, bone, and underlying tissues are the results. Central nervous system injuries as a result of high head acceleration are not to be expected due to blows with beer and/or Coke bottles because of their small weight compared to the head itself (< 0.5 kg vs. approx. 5 kg).

Medical literature shows a variety of tolerance limits of the skin [3, 6, 7, 14–17] and cranial bones [2, 10, 11, 18]. The sometimes high differences in reported fracture thresholds are presumably the consequence of the variability of the used measuring/testing methods and conditions. Research shows that the experimentally determined fracture tolerance of the frontal bone is at 4.0 kN [11], of the occipital bone at 13.6 kN [18], of the parietal bone at 5.8–17.0 kN [2], and of the temporal bone at 6.1 kN [10].

Our results show that a blow with the side aspect of a 0.5-l beer or 0.33-l Coke bottles to an object the size and form of the human skull (and covered with a skin-like, soft layer) reaches a maximum force transmission of approximately 1.3 kN and thus cannot fracture cranial bone.

On the other hand, facial fractures have to be taken into consideration. Twenty-eight out of 162 attempts to shatter the glass reached (exceeded) the 1-kN mark (beer: 6, Coke: 22), which is necessary to fracture the jugual bone [11]. The nasal bone would have suffered a fracture in almost all experiments with its fracture tolerance of 0.5 kN [2]. This supports the statement that medium-sized glass bottles serve as dangerous, albeit not necessarily deadly weapons.

Surprisingly, force transfer of Coke bottles was not higher than the one of beer bottles as much as one might expect from the handling of the two bottles: the Coke glass is thicker, and appears to be more robust and thus capable of causing more severe damage (Fig. 2). However, our experiments have shown that both bottles have comparable fracture thresholds in spite of the thicker glass layer on the part of the Coke bottle.

To cover biological as well as artificial scalp surrogate material, we used both acrylic skin and pork rind. Our study results are in accordance with previously reported lab tests [5, 8, 9]. The higher level of dermal injuries documented in these experiments could be ascribed to the fact that larger (0.75- and 1 l) and heavier bottles were used. Higher mass leads to a higher impulse and, assuming a rather constant impact duration, a higher maximum impact force. Thus, the measured higher maximum force transmission in studies with bigger bottles [13] can be explained.

Based on their lower fracture threshold and therefore minor injury potential, full bottles are expected to transmit a lower maximum force when hitting an object [4], which is the reason why the experiments in this study were conducted with empty glass bottles.

It is important to consider that the abovementioned evaluation applies to medium-sized bottles. Whether larger sized bottles or bottles with thicker glass such as whiskey bottles can cause life-threatening blunt trauma injuries, as proven in our previous study on beer steins [1], will have to be assessed in future experiments.

In the study, regular—used—bottles were tested. Data reported on bar glasses [12] as well as glass beer steins [1] show that brand new pieces of glasswork are more stable and break more easily after use, presumably because of microfractures. This needs to be kept in mind when interpreting the results.

Our results can help to explain the scarcity of severe injuries we observe after assaults with beer or other small bottles to the cranium. They might appear contradictory to the assessment of Bollinger et al. [4] that beer bottles may fracture the human neurocranium because they surpass the minimum fracture threshold. However, measurement conditions must be kept in mind. Whereas Bollinger et al. [4] used energy as a reference parameter; in our study, the impact force was used. Another difference is the impact on a headform (rigid skull model + deformable skin surrogate) in our study, and a steel ball impacting pinewood board fixed to a bottle in the setup used by Bollniger et al. [4]. Furthermore, Bollinger et al. [4] examined the more slender 0.5-l beer bottle type predominant in Switzerland whereas we used the more wider variant predominant in Southern Germany. It must also be taken into account that the headform we used was nondeformable, whereas the human skull deforms prior to fracture. Thus, our measurement setup led to lower contact area and higher stresses (force per area) of the bottles in the contact region and consequently to lower bottle failure thresholds than they would occur in impacts on real human heads. This fact together with the possibility of sturdier (new) bottles does not allow drawing the conclusion from our data that severe head injuries cannot result from blows with beer or coke bottles to the cranium. Also, strikes with other, sturdier bottle parts (the bottom, the neck) could lead to significantly higher force transmissions without a structural failure of the bottle.

## Limitation

The fact that volunteers smashed the bottle on the aluminum dummy head caused a non-standardized impact constellation with several unknown/uncontrolled variables including the (angular) velocity, the mechanical stability of the bottles, and the exact grip on the bottle neck. However, given the nature of circumstances, these variables are also unknown in real-world situations.

Furthermore, given the fact that ballistic test series are based on physical basis, the abovementioned variables are rather secondary. Minor variances of results in forensic assessment of injuries from blows to the head are therefore considered as irrelevant.

The study setup tends to examine a scenario in which the bottles will break more readily than under certain different scenarios. The results at hand refer only to strikes to the vertex of the head as one possible striking area in the context of physical assault with a bottle. Blasts from the side, for instance to the temple, have not been examined. Due to the form of the vertex, namely rounded, this will place a higher strain on the bottle due to a greater energy density. However, other parts of the cranium, such as the temple, which is incidentally very thin and fracture-prone, is a far flatter skull region and the energy density is therefore less. Because of this, the results should carefully be used as comparative figures, when evaluating blasts to the head.

## Conclusion

The possible maximum impact force which can be transferred onto a human skull by blows made with an empty 0.5-l glass beer bottle is comparable to blows with an empty 0.33-l glass Coke bottle.

When striking the vertex of a human adult head, both bottles can cause fractures to the facial bones (esp. nasal bone, zygomatic bone), while cranial bone fractures are unlikely.

If the glass bottle breaks during a blunt assault to the head, the maximum force transmission as well as the risk of blunt trauma is reduced. At the same time, the potential for sharp injuries increases due to the jagged edges of the broken bottle.

Blows with a 0.5-l beer bottle or with a 0.33-l Coke bottle to the head can transfer up to 1.3 kN and thus are able to cause severe blunt as well as sharp trauma injuries. Life-threatening blunt trauma injuries are unlikely in a healthy adult.
